# Continuous Glucose Monitoring under standardised conditions regarding diet, exercise and stress in Healthy Young People (CGM-HYPE study): An exploratory clinical trial

**DOI:** 10.1371/journal.pdig.0001087

**Published:** 2025-11-14

**Authors:** Florian Kinny, Stephanie Läer, Emina Obarcanin

**Affiliations:** 1 Institute of Clinical Pharmacy and Pharmacotherapy, Heinrich-Heine University Duesseldorf, Duesseldorf, Germany; 2 Lee Kong Chian School of Medicine, Nanyang Technological University Singapore, Singapore; University of Reading Reading School of Pharmacy, UNITED KINGDOM OF GREAT BRITAIN AND NORTHERN IRELAND

## Abstract

Continuous glucose monitoring (CGM) in healthy adults is becoming part of healthy lifestyle activities for preventing cardio-vascular and metabolic diseases. However, there is a lack in describing individual glucose responses to everyday situations, with appropriate metrics. The aim of this study was to provide metrics which describe individual glucose responses to lifestyle factors including diet, exercise, and stress in healthy, young adults. Ten participants wore a CGM device (FreeStyle Libre3®) for 14 consecutive days while completing nine standardized interventions (challenges) consisting of food, anaerobic and aerobic sport, and the Trier Social Stress Test (TSST) in an exploratory, clinical trial. Individual glucose responses after each challenge were assessed over four hours, using the following metrics: AUC_0–4_, max glucose, time to max glucose, glucose excursion, and time required for glucose levels to return to baseline (Glucose Recovery Time to Baseline (GRTB)). The study has been registered in the German clinical trial registry (Study number: DRKS00032821). Anaerobic exercise resulted in a significantly greater glucose excursion (28.7 ± 21.46 mg/dL) compared to aerobic exercise (8.8 ± 4.91 mg/dL, *p* = 0.0228). Food with a rich carbohydrate content caused the highest glucose increase (161.4 ± 15.59 mg/dL). TSST resulted in a significant difference in baseline-corrected glucose concentrations over time as revealed by a two-factor repeated measures ANOVA (*p* = 0.0113). We provide reference data of glucose response to lifestyle factors such as diet and exercise in healthy adults. Psychobiological stress revealed a substantial glucose response. Using GRTB metrics may quantify the lifestyle stimulus on the important metabolic pathway and can be utilized alongside kinetic metrics for describing individual glucose responses.

## Author summary

Continuous glucose monitoring (CGM) is widely used in diabetes research but is now gaining more attention for its application in healthy individuals. In this study, we explored how everyday lifestyle factors—diet, exercise, and stress—affect glucose levels in healthy young adults. Over two weeks, ten participants wore CGM devices while completing controlled challenges, including consuming specific foods, performing aerobic and anaerobic exercise, and undergoing a stress-inducing task. We observed that anaerobic exercise caused greater glucose spikes than aerobic exercise, while carbohydrate-rich foods triggered the highest glucose increases. Psychological stress also led to significant glucose changes, underscoring its impact on metabolic health. To analyse individual responses, we introduced the Glucose Recovery Time to Baseline (GRTB) metric, which measures the time for glucose levels to normalize after a challenge. Our findings provide valuable reference data on glucose responses to lifestyle factors in healthy adults. This research highlights CGM’s potential for tracking glucose dynamics in non-diabetic populations and offers new insights into how lifestyle influences metabolic health, paving the way for personalized strategies to prevent metabolic and cardiovascular diseases.

## Introduction

Over the past 20 years, continuous glucose monitoring (CGM) has evolved from rudimentary sensors to advanced devices that monitor glycaemic status. Today, accurate, minimally invasive sensors are available that can be worn on the upper arm for up to 14 days to electrochemically measure glucose in interstitial fluid (ISF) [1,2] generating glucose profiles displayed on an external smart device [3,4]. In contrast to manually self-monitoring blood glucose (SMBG), CGM systems do not require blood samples and can provide glucose values more conveniently while simultaneously gathering far more data. Another advantage is the possibility to measure glucose levels even during times of exercise, swimming, work or sleep [5–7].

Meticulous monitoring of glucose levels is essential for a wide range of users. Initially, this technology was designed for individuals requiring management of their pathological glucose levels. This encompasses patients with type 1 diabetes mellitus (T1DM), type 2 diabetes mellitus (T2DM), gestational diabetes, as well as those diagnosed with pre-diabetes or metabolic syndrome [8,9]. In these cases, CGM systems are used in clinical settings to manage therapy and to monitor disease progression as an essential part of disease management. Its data can be used to improve disease markers and adapt therapy accordingly.

CGM systems, however, have been used far beyond diabetic disease management nowadays. By providing information about physiological variables, CGM systems are employed in athletic contexts to optimise peak physical performance, to monitor for hypoglycaemia, and to refine or to individualize training strategies [10]. Moreover, due to providing direct biofeedback, CGM devices as well as devices such as smartwatches are used by healthy individuals to monitor glucose levels and to record vital signs, i.e., sleep cycles and heart rate, in order to establish or maintain a healthy lifestyle. Insight into real-time glucose values can effectively motivate individuals to adopt lifestyle choices that enhance healthy dietary habits, support weight management, increase physical activity, and improve stress regulation [11,12]. Wearable devices are regarded as valuable tools in these efforts [13–15].

The widespread use of CGM requires a more comprehensive knowledge of factors stimulating glucose levels in everyday life. This includes already known biochemical and physiological processes but, moreover, it requires additional research into new connections. It is well-known that hormonal fluctuations, particularly during menstruation or menopause can impact glucose dynamics [16,17]. Additionally, factors such as age [18], gut microbiome [19,20] and genetic factors affecting ß-cell performance [21] have been identified as contributors to glucose regulation. Lifestyle factors, including sleep quality [22], stress levels [23,24], smoking habits [25], and physical activity [26], also play a crucial role in glucose dynamics. Dietary factors significantly influence glucose levels, with the composition of food [27,28] as well as the timing and order of consumption being crucial elements [29–31]. Physical activity likewise has a strong impact on glucose levels [32,33]. While effects of diet and exercise on glucose levels are well-known, data on the impact of psychobiological stress on glucose are limited but vital for reducing the risk of chronic diseases.

To understand the effects of diet, physical activity and stress regarding glucose dynamics, standardized methods to describe individual glucose levels are needed. CGM data are currently presented in a comprehensive manner in the ambulatory glucose profile (AGP) [34,35] displaying pre-defined metrics like average glucose, glucose variability, or time in ranges to quantify and assess glycaemic status [36,37]. Studies involving healthy individuals wearing CGM devices without further intervention [38–40] aimed to collect reference data in accordance with international consensus. While these metrics are primarily utilized to assess diabetes management, they offer limited utility for individuals without diabetes. This limitation arises from the aggregation of all data points over the entire 14-day monitoring period, which precludes the assessment of individual glucose responses to specific stimuli, including meals, physical activity, or stress—factors that may be of significant interest. To effectively quantify individual glucose peaks, alternative metrics are necessary including the area under the curve of a glucose peak (AUC), the maximum peak glucose value (c_max_), glucose excursion (difference between c_max_ and baseline), or the time taken to reach the peak (t_max_). Notably, a method to quantify the duration required to return to initial baseline glucose levels (Glucose Recovery Time to Baseline, GRTB) is currently absent, yet this information could be crucial for evaluating the body’s capacity to manage glucose effectively. The indication of the initial time point at which glucose returns to baseline levels does not sufficiently address potential fluctuations or postprandial and post-exercise hypoglycaemia. Existing studies have examined individual glucose profiles following standardized interventions, predominantly focusing on diet using those metrics [29–31,41]. However, there remains a gap in research describing the effects of standardized physical activity and stress on glucose dynamics using metrics to quantify individual glucose dynamics.

Therefore, this research aims to conduct a standardized analysis of CGM data obtained from healthy young adults under controlled conditions, with a focus on individual glucose responses due to diet, physical activity and psychobiological stress, to establish these data as reference values.

## Methods

### Ethics statement

This study was conducted in accordance with the Declaration of Helsinki and was approved by the Ethics Committee of the Faculty of Medicine, Heinrich-Heine University Duesseldorf (Study number: 2023–2647). It has been registered in the German clinical trial registry (Registration number: DRKS00032821). Prior to participation, informed consent was obtained from all subjects involved in this study.

### Study design

A one-centre, single-arm, exploratory, interventional, clinical trial involving healthy, young adults was conducted between April – July 2024 at the Institute of Clinical Pharmacy and Pharmacotherapy, Heinrich-Heine University Duesseldorf, Germany. Participants were recruited through personal networks as well as on the university campus. Inclusion criteria included: ≥ 18 and <40 years old, body mass index (BMI) ≥18.5 and <30 kg/m^2^, waist circumference (men ≤ 94 cm, women ≤80 cm), fasting blood glucose (FBG) <100 mg/dL, blood glucose <140 mg/dL two hours after oral glucose tolerance test (2h-OGTT), possession of a mobile device and willingness to use it, voluntary consent to participate in the interventional study and written consent to data protection in the context of the interventional study.

Participants diagnosed with T1DM or T2DM, smokers, pregnant women, those with cognitive or physical impairment, eating disorders, and any chronic disease affecting glucose metabolism, were excluded, as well as participants taking drugs with metabolic effects (e.g., glucocorticoids).

### Intervention

After the prospective study participants were able to be enrolled in the study, they were informed about the purpose and procedure of the study in an informational seminar. A CGM device (FreeStyle Libre® 3, Abbott Diabetes Care GmbH, Wiesbaden, Germany) was attached to each participant’s upper, non-dominant arm, according to manufacturer’s instructions, and was worn for up to 14 days. The accuracy of the CGM is reported as a mean absolute relative difference (MARD) of 7.8% [42]. ContourNext® glucometers (Ascensia Diabetes Care Deutschland GmbH, Leverkusen, Germany) were used to test FBG and 2h-OGTT during recruitment.

### Study procedure

**Fig 1:**
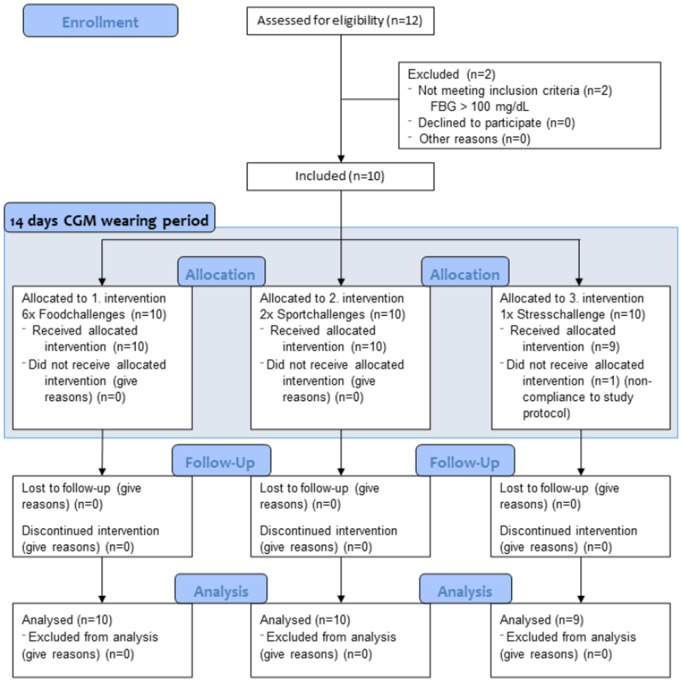
CONSORT Flow Diagram (CGM-HYPE-Study). FBG = Fasting Blood Glucose, CGM = Continuous Glucose Monitoring.

The study duration for each participant was 14 days (Fig 1). Participants were asked to document the time of intake and the amount of carbohydrates (CHO), proteins, and fats consumed. Physical activity specified by the type of activity was documented including the activity duration and intensity. Prior to data generation, an introductory seminar was held by the study coordinator for all participants to minimize potential self-reporting bias. During this seminar, the study objectives and procedures were clearly explained, and participants received standardized instructions on documenting their dietary intake and physical activity. After 14 days, study staff removed the sensors and examined the participants’ skin for irritation. Participants were asked to complete three questionnaires on CGM utilization, usefulness, experience with the sensor, and their quality of life (QoL) during the trial. No financial remuneration was offered to participants for their involvement in the study.

The individual glucose response to each challenge was analysed by determining:

(1) initial baseline (mean of the three consecutive glucose readings recorded before the start of the challenge)
(2) area under the glucose curve (AUC) from the start of the challenge to four hours after the challenge using trapezoid rule
(3) c_max_: maximum glucose concentration
(4) t_max_: time to c_max_
(5) glucose excursion (difference of the c_max_ and initial baseline)
(6) peak duration as time required for glucose levels to return to initial baseline (Glucose Recovery Time to Baseline (GRTB))

To isolate the individual glucose profile of a respective challenge, participants were instructed to refrain from eating and exercising for four hours before and for four hours after each challenge.

### Food challenge

Each participant consumed in total six prepared standardized meals consisting of two different breakfasts (F1,F2) and two different lunches (F3,F4), repeating one breakfast (F2) and one lunch (F3) once. The composition and quantity of food for the food challenge can be found in S1 Table. Participants received detailed, written instructions on the exact timing and manner of consuming the food (S2 Table).

### Sport challenge

Two physical activity units had to be carried out: 1) a 40-minute functional fitness aerobic training with 12 exercises using own body weight following written instructions (PA1) (S3 Table) and 2) a 30-minute endurance anaerobic training on an aerial bike (PA2). For this purpose, the maximum load threshold was established for each participant [43]. The resistance was incrementally increased by 40 watts at four-minute intervals until the individual’s maximum was attained. This resistance level was subsequently maintained for the remainder of the duration.

### Stress challenge

The standardized protocol of Trier Social Stress Test (TSST) [44] was conducted between 3:00 pm and 5:00 pm, in both a test and a control session, to induce psychobiological stress through a combination of social evaluative threat and uncontrollability including a four minute mock job interview and a surprise four minute arithmetic task (subtracting 16 from a four-digit number) in front of a panel of two (S1). The panel, wearing white coats and introduced as behavioural research experts, was instructed to provide no verbal or non-verbal feedback during the sessions. Two dummy cameras were set behind the panel. After ten minutes, the test ended and participants were led to another room and asked to relax for 30 minutes. Seven days later, the test was repeated in controlled settings, eliminating the element of social evaluation while maintaining other aspects of the procedure as closely as possible. Additionally, the panel did not wear lab coats during the control session. Instead of a job interview, participants were asked to talk about a friend. The arithmetic task was counting from 0 in steps of five. To objectively assess the effect of the stress test, saliva samples were taken from the participants five minutes before the test (minute -5), after each task during the test (minutes 5 & 10), and 30 minutes after the stress test (minute 40) to analyse the cortisol levels as a stress marker [45]. The saliva samples were collected using Salivette® (Sarstedt AG & Co. KG, Nümbrecht, Germany). Each Salivette® contained a woolen tube, on which the participants were asked to lightly chew for 60 seconds. The Salivette® were stored at -20°C. All samples were sent to laboratory (Dresden LabService GmbH, Dresden, Germany) for analysis by luminescent immunoassay (IBL international, Hamburg, Germany). Furthermore, participants’ subjective stress level was measured at the same time as the cortisol using a Visual Analogue Scale (VAS) to indicate how stressed participants felt on a scale from 0 (not stressed at all) to 100 (very stressed).

While the stress and anaerobic sport challenges were conducted on-site at the institute under supervision by study personnel, the remaining challenges were self-administered by participants at home without direct supervision. To ensure consistency and adherence, detailed instructions for these home-based challenges were provided during a prior seminar and supplemented by an instructional handout given to each participant.

### Questionnaire

A seven-point Likert scale was available for rating the handling of a CGM (1 = “strongly disagree”, 7=”strongly agree”). Usefulness was rated using a five-point Likert scale (1 = ”not at all useful”, 5 = ”extremely useful”). Participants were asked to use a slider (0 = very bad,100 = very good) to indicate the usefulness of viewing their glucose levels continuously and to rate their experience with a CGM using a trichotomous scale (“yes”,”neutral”,”no”). In addition, the WHO-5-Well-being index questionnaire [46,47] was used to assess participants’ QoL in the last two weeks.

### Statistical analysis

Results are expressed as mean ± standard deviation (SD) or median (interquartile range (IQR)) depending on the distribution of variables. Glucose metrics exceeding IQR times 1.5 were seen as an outlier and therefore excluded from the analysis. The percentage of time spent within a given threshold was determined by dividing the number of CGM readings that were within the threshold by the total number of CGM readings recorded by the participant. A paired t-test was used to compare the cortisol at baseline and 30 minutes after the stress and to compare the mean glucose values one hour before and one hour after stress induction. To compare mean glucose concentrations before the stress induction between the control group and the intervention group and to compare CGM metrics, Mann-Whitney test was used. A two-factor repeated-measure analysis of variance (ANOVA) with Greenhouse-Geisser correction and Bonferroni testing was performed to determine differences in glucose levels, VAS scores, and cortisol concentrations over time. To test post-hoc which timepoints regarding glucose significantly differed after inducing stress, a two-way ANOVA with repeated measurements and with multiple comparison was performed. To account for multiple comparisons, we applied a Bonferroni correction to all statistical analyses. As a result, significance was determined using an adjusted p-value threshold of 0.05.

Data was maintained using Excel [48] and python programming language [49]. The data was analysed using the R programming language [50] and OriginPro® [51]. QualtricsXM® software [52] was used to create and administer the electronic questionnaires.

A hypoglycemic episode starts when sensor glucose readings remain below 54 mg/dL for 15 minutes or more. The episode is considered over only when glucose levels stay at or above 70 mg/dL for at least 15 consecutive minutes. GRTB was determined by calculating the SD of three consecutive glucose values. If none of three consecutive SD exceeded 2.5 mg/dL, the timepoint of the second glucose value used to calculate the second SD was taken as the time to reach baseline, provided this value did not deviate from the initial baseline by more than 2.5 mg/dL. A margin of 2.5 mg/dL was set based on the baseline SD in this healthy population. The time period between 06:00 am to 11:59 pm was considered as daytime and 12:00 am to 05:59 am as night time [53]. To investigate a potential dawn effect (natural rise in glucose that occurs in the early morning hours), glucose levels at 3:00 am and 5:00 am were compared to 1:00 am, by calculating the mean glucose values 15 minutes before to 15 minutes after each timepoint. Glucose values in mg/dL can be converted to mmol/L by dividing by 18.02.

## Results

### Participants/ baseline data

A total of 12 people were screened for this exploratory study. Two participants were excluded due to FBG levels exceeding 100 mg/dL at time of recruitment (Fig 1). Eventually, ten participants were included in the exploratory feasibility study after having provided informed written consent. Table 1 shows the baseline characteristics of the study cohort.

**Table 1:** Baseline participant characteristics.

Characteristics	Overall	Male (n = 4)	Female (n = 6)
Age, years (mean ± SD)	29 ± 3.77	31.25 ± 4.32	27.5 ± 2.36
18–24 n (%)	1 (10)	--	1 (16.67)
25–29 n (%)	4 (40)	1 (25)	3 (50)
30–34 n (%)	4 (40)	2 (50)	2 (33.33)
35–40 n (%)	1 (10)	1 (25)	--
Range	23 – 38	26 – 38	23 – 30
Waist circumference (mean ± SD)	74.9 ± 7.43	82.5 ± 3.77	69.83 ± 4.3
Hip circumference (mean ± SD)	90.5 ± 9.78	93.75 ± 3.34	88.33 ± 11.84
BMI, kg/m^2^ (mean ± SD)	22.1 ± 2.62	23.36 ± 2.66	21.26 ± 2.24
Range	18.47 – 27.77	20.68 – 27.77	18.47 – 25.28
BMI category^a^, n (%)			
Underweight	1 (10)	--	1 (16.67)
Normalweight	8 (80)	3 (75)	5 (83.33)
Overweight	1 (10)	1 (25)	--
2h-OGTT, mg/dL (mean ± SD)	114.2 ± 21.77	104.75 ± 27.4	120.5 ± 13.78
FBG, mg/dL (mean ± SD)	92.8 ± 3.92	91.5 ± 4.72	93.67 ± 2.98

SD = Standard deviation, BMI = Body-Mass-Index, 2h-OGTT = 2h blood glucose value during an oral glucose tolerance test, FBG = fasting blood glucose.

^a^The BMI categories for underweight, normal weight, and overweight for participants aged >18 years are < 18.5, > 18.5 to <24.9, and >24.9, respectively.

### Outcomes

#### Glucose metrics.

Glucose data over 24 hours and on different times of day are shown in S4 Table. No hypoglycaemic events were detected during the entire wearing period. The mean of all glucose data during the day was 107.57 ± 7.22 mg/dL and 102.26 ± 5.72 mg/dL during the night. The mean value for women was 103.09 ± 6.43 mg/dL and 110.96 ± 2.61 mg/dL for men. As a measure of glucose variability, a coefficient of variation (CoV) of 17.13 ± 3.54% was determined for the daytime and 12.98 ± 2.96% for the nighttime. CoV at night were similar for women and men.

#### Challenge.

**Fig 2:**
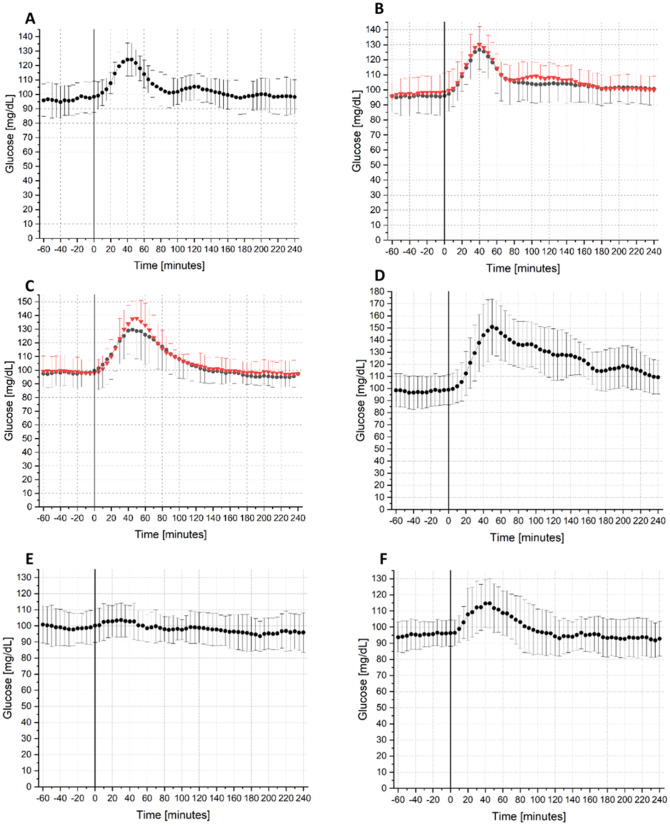
Mean glucose concentrations ± standard deviation (SD) measured by a Continuous Glucose Monitoring (CGM) device during food (A-D) and sport (E-F) challenge. Start of challenge at minute 0, each n = 10 (A) Breakfast challenge without additional fibre (B) Breakfast challenge with additional fibre, conducted twice (C) Lunch challenge conducted twice (D) Lunch challenge Pizza (E) aerobic sport challenge (F) anaerobic sport challenge. The red lines represent the second trial of challenges that were all conducted twice.

Table 2 shows the mean values and standard deviation of glucose metrics across all participants for each challenge. Outlier exclusion was performed at the level of individual glucose metric values within each challenge, not at the subject level. Specifically, values exceeding 1.5 times the IQR were considered outliers and excluded from further analysis. Overall, this procedure resulted in the exclusion of 3.37% of the data points. Fig 2 presents the mean glucose levels for all participants after the food and sport challenges, together with the respective standard deviations.

**Table 2:** Results of CGM-metrics for each challenge.

Mean (± SD)	Baseline [mg/dL]	c_max_ [mg/dL]	t_max_ [min]	GRTB [min]	Excursion [mg/dL]	AUC_0-4h_ [min*mg/dL]
F1 Breakfast 1 without fibre	97.97 (±11.01)	123.22 (±7.79)	42.22 (±6.67)	128.75 (±34.92)	28.13 (±13.9)	25084.5 (±2055.51)
F2.1 Breakfast 2 with fibre	95.77 (±12.46)	131 (±11.29)	44.5 (±9.34)	135.71 (±68.7)	35.23 (±12.79)	25438 (±2467.35)
F2.2 Breakfast 2 with fibre	98.33 (±11.61)	133.1 (±11.87)	41.5 (±7.09)	159.38 (±38.4)	36.44 (±4.94)	25803 (±1882.98)
F3.1 Lunch 1 Potatoes with vegetables in curry sauce	101.22 (±7.05)	142.78 (±12.28)	48.5 (±11.84)	149.17 (±36.93)	39.97 (±12.15)	26218.61 (±1137.66)
F3.2 Lunch 1 Potatoes with vegetables in curry sauce	97.8 (±9.66)	142.8 (±15)	49.5 (±10.11)	137.5 (±44.81)	45 (±14.29)	25590.83 (±1086.19)
F4 Lunch 2 Pizza Margherita	100.43 (±5.36)	161.4 (±15.95)	52.22 (±12.77)	142.5 (±53.03)	62.93 (±18.28)	29999.25 (±2917.6)
PA1 Aerobic functional fitness	98.7 (±9.87)	107.5 (±9.51)	25.00 (±15.58)	73.33 (±57.61)	8.8 (±4.91)	23567.5 (±2225.28)
PA2 Anaerobic endurance training	96.2 (±7.98)	124.9 (±17.37)	39 (±24.37)	117.14 (±53.3)	2.7 (±21.46)	23175 (±1068.01)
S1 Stresstest: TSST	95.56 (±11.43)	102.5 (±11.71)	37.5 (±11.34)	104.29 (±74.91)	9.67 (±7.13)	23167.86 (±2059.47)

SD = Standard deviation, AUC = Area under the curve, mg = milligram, dL = decilitre, min = minutes, TSST = Trier Social Stress Test, GRTB = Glucose Recovery Time to Baseline.

Mean basal value of 97.45 mg/dL ± 1.24 mg/dL was calculated before each challenge. With the most CHO, pizza resulted in the largest AUC and glucose excursion, as well as the largest c_max_. The average c_max_ for breakfasts was 130.16 ± 12.14 mg/dL. The lunches achieved an average c_max_ of 147.93 ± 19.14 mg/dL. Mean glucose value at night, which followed the daytime sport challenge, was 105.12 ± 8.27 mg/dL and 103.04 ± 6.34 mg/dL for aerobic and anaerobic training respectively. Aerobic and anaerobic training significantly differ regarding c_max_ (*p* = 0.0411) and glucose excursion (*p* = 0.0168). Similar glucose profiles were observed when comparing identical food challenges. Adding additional fibre to breakfast meals resulted in a higher peak glucose value compared to breakfast without added fibre, with the peak occurring at similar times. While the average glucose profile after aerobic exercise is flat, the glucose profile after anaerobic exercise shows a rise and course comparable to that of a breakfast. No dawn effect was detected in this cohort, at neither 3:00 nor 5:00 am. The GRTB could not be determined for all participants in every challenge.

**Fig 3:**
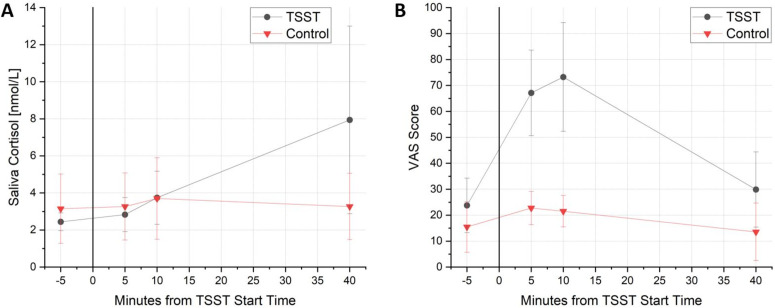
Mean values with SD of nine participants at four different measurement time during TSST for A) salivary cortisol and B) subjectively reported stress levels via VAS. TSST took place between minute 0 and minute 10. SD = standard deviation, TSST = Trier Social Stress Test, VAS = Visual Analogue Scale.

A total of nine people took part in the TSST, both in test and control session. Each wore a CGM during the test conditions. During the control session, glucose data could only be gathered from five people. One participant was excluded from the analysis due to non-compliance with the study protocol. Due to the limited number of observations in the TSST condition, we restricted our analysis to the 60-minute periods immediately before and after the stressor. This adjustment was made to maximize the available data and ensure the robustness of our findings for this condition. Mean salivary cortisol concentrations significantly increased from 2.44 ± 0.48 nmol/L baseline to 7.94 ± 5.06 nmol/L 30 minutes following the TSST (*p* = 0.0188). On control day mean salivary cortisol concentrations were 3.15 ± 1.87 nmol/L and 3.27 ± 1.79 nmol/L, at baseline and 30 minutes (*p* = 0.7908) respectively (Fig 3A). There was no significant difference between salivary cortisol concentrations at baseline between test and control sessions (*p* = 0.3085). The two factor ANOVA with repeated measurements revealed peak cortisol concentration in stress session at minute 40 being significantly higher compared to concentrations of each measurement timepoint before. VAS scores were significantly higher during the test session compared to baseline (Fig 3B).

**Fig 4:**
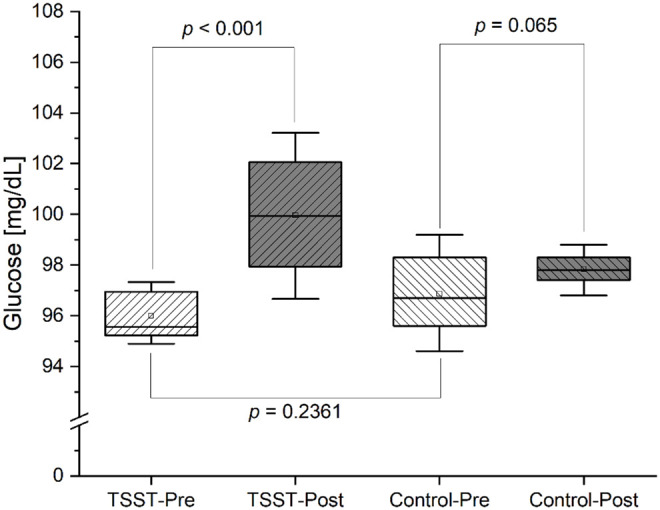
Comparison of mean glucose values 60 minutes before and after the Trier Social Stress Test (TSST) in stress and control session. The hollow rectangle represents the mean value of the data. A paired t-test was used to compare Pre- to Post-data. The comparison of Pre-TSST with Pre-Control was carried out using the Mann-Whitney test. A significance level of 0.05 was applied in each case.

**Fig 5:**
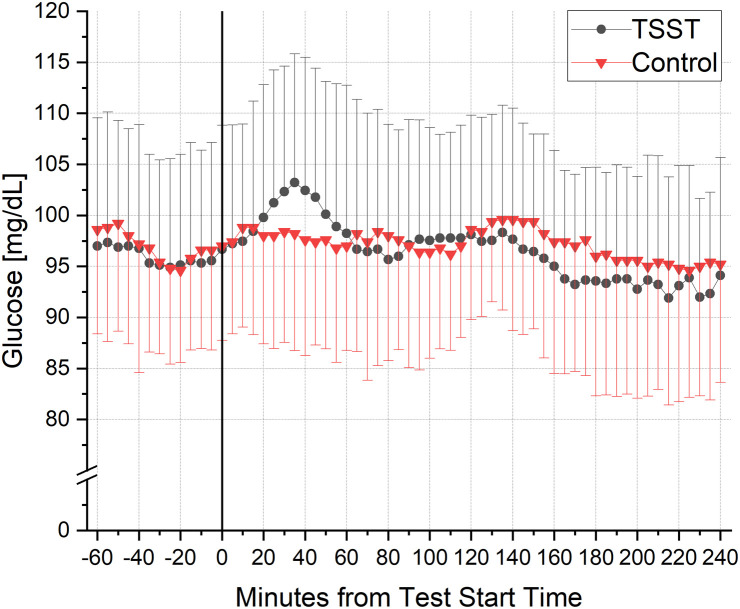
Glucose concentrations ± standard deviation (SD) measured by a Continuous Glucose Monitor (CGM) under stress conditions (Circle, n = 9) and under control conditions (Triangle, n = 5). TSST took place between minute 0 and minute 10. TSST = Trier Social Stress Test.

AUC of baseline-corrected cortisol increased in the stress group, but not in the control group (*p* = 0.0411) (data not shown). A two-factor ANOVA with repeated measurements revealed a significant difference in the course of baseline-corrected glucose over time (*p* = 0.0113). Post-hoc multiple comparison analysis showed a significant difference between the two conditions (TSST x Control)(*p* = 0.0112). On stress day, mean glucose value 60 minutes before the start of the challenge was significantly higher than 60 minutes after the start of the stress (*p* < 0.001) (Fig 4). Glucose profiles on stress and control session are presented in Fig 5.

### Questionnaire

**Fig 6:**
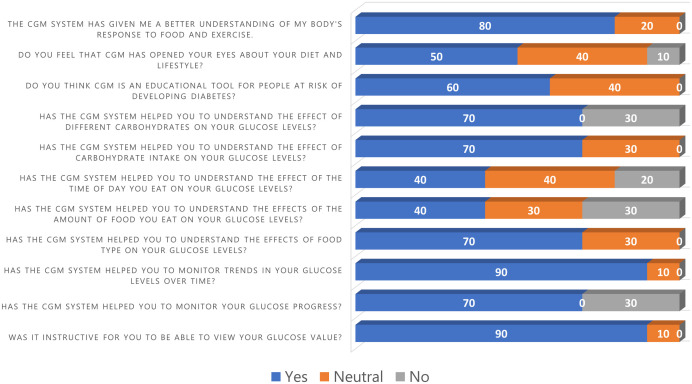
Stacked Bar plot of participants’ experience with a continuous glucose monitoring (CGM) system, n = 10.

Participants indicated a high level of satisfaction with CGM and stated they consider the device user-friendly (S1 and S2 Figs). CGM was found to be helpful in assessing the influence of food and its composition on glucose profile. With the help of CGM, a better understanding of the body’s response to food and exercise was achieved (Fig 6). In addition to the quantitative data presented in Fig 6, participants in general reported that the CGM system was useful and easy to use in every-day life. Most participants reported that receiving real-time glucose feedback helped them understand how different activities and meals influence their individual glucose responses. Furthermore, the majority found that the device was comfortable to wear and stated that integrating CGM measurements into their daily routine was manageable.

While wearing the CGM device, the QoL was rated as high by the WHO-5 (S3 Fig).

### Adverse events

None of the participants reported experiencing adverse effects (0/10, 0%).

## Discussion

In this study, standardized interventions regarding diet, exercise, and stress were carried out to examine individual glucose responses with corresponding kinetic metrics in healthy, young people which can serve as reference of CGM glucose profiles for future studies, while providing valuable insights into personalized glucose management. We used kinetic-mathematical metrics to describe individual glucose responses to specific stimuli and present a method to quantify GRTB. A high carbohydrate load led to highest c_max_ and long glucose peak. During anaerobic training, glucose levels increased, whereas glucose levels remained relatively steady during aerobic training. The induction of stress caused glucose to rise significantly compared to a control setting. Participants reported a high level of QoL during the 14-day wearing period while stating satisfaction with the handling and usability of a CGM device which helped participants to understand the impact of diet, exercise and stress on their glucose levels. CGM was perceived both as educational and beneficial, suggesting its applicability for lifestyle-monitoring among healthy individuals.

The results of this investigation are in line with CGM values reported in the literature for healthy participants, as described by available CGM metrics. The mean glucose value of the study population was 106.24 mg/dL (mean CoV of 15.65%) with median time in a glucose range of 70–140 mg/dL of 96%, which by far exceeds the international consensus target of 70% for people with diabetes [36]. No values below 54 mg/dL or above 250 mg/dL were recorded, indicating the absence of hypoglycaemic events. Compared to daytime, mean night glucose values were lower, and without prominent nightly glucose fluctuations. These data are consistent with previous studies which showed that values above 140 mg/dL rarely occur in healthy individuals [38–40,54]. These data were collected solely through the use of CGM systems without additional interventions and provide reference data in the form of standard CGM metrics in accordance with international consensus. Our data supplement these data and can be used as a threshold for healthy adults in further research.

### Glucose dynamics

New metrics are required to describe individual glucose reactions, since available metrics only summarize the entirety of data. For this purpose, kinetic-mathematical metrics such as AUC, c_max_, t_max_, initial baseline and glucose excursion have been utilized to describe postprandial glucose dynamics [29,30,41] and exercise-related glucose responses [32]. As GRTB reflects the body’s ability to maintain glucose homeostasis, it represents an important metric for biofeedback for healthy individuals or in the context of disease management for diabetes patients. However, the investigation of GRTB remains underexplored. It is not yet mentioned in medical guidelines and is currently defined inconsistently. Existing approaches of quantifying GRTB, such as assessing recovery percentages [55] or defining hyperglycaemia duration [56], fail to account for glucose fluctuations or complex response curves. To address this, we propose calculating GRTB using SD of three consecutive glucose values to define baseline stability, or alternatively, using rate of change (ROC). Three values seem to be sufficient as it is used to calculate baseline [32] or in meal detection algorithms [57]. While this approach is promising, high glucose variability within a four-hour observation period occasionally precluded GRTB determination. These cases were excluded from the GRTB-based analyses for the respective conditions, where glucose levels did not return to baseline values within the observation timeframe. As a result, the sample size for GRTB comparisons varies across conditions. We acknowledge that this exclusion may introduce bias, as participants exhibiting prolonged glucose recovery are not represented in the GRTB-based comparisons. To avoid this in future research, an extended observation period could be provided. Nonetheless, these findings offer valuable preliminary insights into the glucose recovery dynamics under various challenge conditions. Since high glucose variability, low percentage of time in the target range (TIR) and postprandial hyperglycaemia are associated with an increased risk of diabetic complications [58–61], it can be assumed that short GRTBs are generally advantageous. The assumption that postprandial glucose levels return to the initial baseline within 2–3 hours [62,63] is supported by our data. Using the proposed method, GRTB can now be quantified as an endpoint.

### Food

Postprandial glucose responses after standardised meals were investigated using new metrics. As expected, pizza containing a high amount of CHO resulted in the highest AUC [64], with two participants exhibiting glucose levels exceeding 180 mg/dL, despite the high fat content, which has the potential to flatten postprandial glucose response [65]. These findings are in line with the literature [29]. Our data corroborate previous findings, indicating that c_max_ occurs approximately 40–50 minutes after food intake [30]. Dietary fibres affect glucose response by delaying digestion of CHO, which may extend glucose absorption [66]. This is supported by the increased GRTB observed in study breakfasts with additional fibre. Given that the sequence and timing of food intake significantly influence postprandial glucose levels, both factors were standardized in this study [67,68]. Despite standardization of the interventions, it is crucial to acknowledge the presence of variability [69]. The dual implementation of two challenges enables direct comparisons. In this context, participants exhibited comparable glucose dynamics, with no significant deviations observed in the metrics used to characterize individual glucose curves.

### Sport

While intensive anaerobic training caused an average increase in glucose, it remained relatively steady during aerobic training, with significant differences observed in c_max_ and glucose excursions. DuBose et al. observed a decrease in glucose during both anaerobic and aerobic training [32], describing individual glucose dynamics using c_min_ and glucose excursion. However, comparability is limited due to lack of standardization. In contrast to the data of DuBose, no hypoglycaemic event was observed after an exercise session in the presented data. Nocturnal glycemia did not show any clinically differences between exercise and non-exercise days. This observation is in agreement with findings reported in the literature [32].

### Stress

New metrics and CGM were applied to describe glucose dynamics following induction of stress. These metrics revealed a modest and short-lived increase in glucose levels. To induce psychobiological stress, commonly used standardized TSST was used [44], which is described in the literature [70–75]. The physiological and biochemical mechanisms behind the glucose increase are well-known. TSST has been shown to enhance sympathetic activity [76], inducing a stress response that activates the hypothalamic-pituitary-adrenal (HPA) axis. This activation releases cortisol from the adrenal cortex subsequently producing insulin resistance [24] and stress hyperglycaemia through multiple mechanisms. First, it stimulates gluconeogenesis in the liver and provides amino acids as precursors through its catabolic effects. Additionally, it inhibits the conversion of glucose into glycogen in both liver and muscles. Moreover, cortisol suppresses lipogenesis. Lastly, cortisol, in conjunction with ACTH, activates the adrenal medulla to release catecholamines, which further elevate glucose levels by enhancing glycogenolysis [77,78]. In T1DM patients, however, contradictory evidence has been reported so far. In 2005, Wiesli et al. [80] reported an increased insulin resistance in T1DM patients after meal consumption following TSST protocol. The authors did not observe a significant increase in glucose levels using CGM. In contrast, Kaur et al. [79] provided indirect evidence for an increased glucose response to TSST reporting about a sensor-augmented pump (SAP, mean glucose = 158.1 mg/dL, mean c_max_ = 246.5 mg/dL, N = 12) and automated insulin delivery (AID, mean glucose = 150.0 mg/dL, mean c_max_ = 227.4 mg/dL, N = 12). Using CGM and a new metrics analysis method, to our knowledge, we present comprehensive CGM data after conducting a TSST in healthy, young adults for the first time. These data not only provide insights into the physiological responses to acute stress but may also serve as a foundation for further studies exploring stress monitoring in real-life contexts, such as work-life balance in free-living individuals. Given that chronic stress contributes to glycaemic dysregulation, our findings offer a valuable contribution to diabetes research. By building on this preliminary work, future research can validate and expand upon our findings to establish broader conclusions by incorporating a larger sample size as well as additional objective parameters, such as heart rate measurements, to enhance the robustness of findings.

### Survey

Participants stated a comfortable use of CGM device and the associated app, an easy sensor application and navigation within the app. These findings suggest that CGM systems are not only feasible for use in research context but also well accepted by users in real-world setting, supporting their suitability for wider adoption in everyday personal health monitoring. These results are in agreement with previous studies [80,81]. Since prior investigations have shown that CGM is regarded as helpful in encouraging more mindful eating and greater physical activity [82–86], our findings support the idea that CGM as a wearable device can be easily incorporated into daily life and can function as an effective tool for lifestyle monitoring. Real-time feedback and heightened awareness of the impact of various foods on glucose levels appear to support positive changes in nutritional behaviour and may also serve as a motivating factor for promoting regular physical activity [11,84,87].

This study has several notable strengths. Firstly, it focuses on a homogeneous population of young, healthy adults, which enhances the reliability of the findings by excluding those with (pre)diabetes. This might be of interest for healthy adults using CGM to monitor their lifestyle. Secondly, the introduction of GRTB as a novel metric for analysing individual glucose responses offers valuable insights into the mechanisms by which the body regulates glucose homeostasis, helping to understand the complex interplay involved in maintaining stable glucose levels. Additionally, the use of a modern, more accurate RT-CGM-device [42] is an advantage compared to research using older flash glucose monitoring (FGM) devices with risk of data loss due to missed scanning. In contrast to other studies where participants were only required to wear CGM devices for the assessment of standard CGM metrics, our research enabled to investigate individual glucose responses through standardized interventions. This approach allowed for a more detailed analysis of how specific interventions affect glucose levels, providing valuable insights into personalized glucose management. Finally, the collection of CGM data from non-diabetic people undergoing TSST as a standardized stress test, which incorporates both subjective as well as objective measures to evaluate the induced stress, enhances the depth and rigor of our analysis. This comprehensive approach allows for a more nuanced understanding of physiological responses to stress, contributing valuable insights into the interplay between stress and glucose regulation.

Despite the strengths of this study, it also exhibits certain limitations. A key limitation of our study is the small sample size (n = 10), which substantially limits the statistical power and generalizability of the findings. As a result, these findings should be interpreted with caution, when considering their extrapolation and applicability a to broader populations. Future research should involve larger and more diverse cohorts to validate and extend these preliminary findings, thereby increasing the robustness and applicability of the results. The sample size of n = 10 was chosen based on practical considerations, due to the intensive, time and cost-demanding nature of the study protocol, including CGM, detailed dietary, physical activity and stress-exercise monitoring. Furthermore, as an exploratory study, the primary objective was to assess feasibility and to capture variability in individual glucose, rather than achieving a generalization in larger population. This approach is consistent with similar CGM based studies, which employed a small sample size, similar to this study [40,88], producing meaningful insights. These studies demonstrate that even with a limited number of participants, when employing intensive and multidimensional data collection, meaningful insights into glucose dynamics can be obtained. These findings can be useful and inform future larger-scale research. To minimize potential self-reporting bias, all participants attended an introductory seminar prior to data generation. During this session, the study objectives and procedures were explained in detail and participants received specific instructions on documenting their dietary intake and physical activity. Despite these measures, we acknowledge that self-reporting bias remains a possible limitation of our study. Future research should consider the use of more objective tools, such as detailed dietary logs, electronic food diaries, or activity trackers, to further reduce this bias and enhance data accuracy. An additional limitation of this study is the reliance on self-reported healthy status without measured HbA1c testing. Participants’ normal glucose management indicator (GMI) values can be referenced in this context. While our study provides important exploratory insights, the relatively homogeneous sample limits the generalizability of the findings. Future studies should include participants with a broader range of ages, ethnicities, and health backgrounds to better capture the diversity of a representative healthy population. Although only salivary cortisol was objectively measured during TSST, it has proven effective and sufficient without the need to determine blood cortisol levels [89]. Additionally, subjective parameters were assessed using VAS, which helps to compensate for this limitation. The reduced sample size in the TSST control group due to participants not adhering to the study protocol (e.g., eating four hours prior to start of protocol) was addressed by analysing the mean glucose levels, which were available for the nine-participant sample. This approach enabled the extraction of meaningful insights from the available data, even with a smaller sample size. Furthermore, it is also worth noting that four glucose profiles could not be analysed due to non-compliance with the study protocol during TSST. Moreover, future studies need to focus on dinner as a meal time, as the timing of food intake influences the glucose response due to shifted insulin sensitivity. Lastly, while BMI was used as a measure, its limitations—stemming from its derivation from earlier non-Hispanic population data—were addressed by including waist circumference measurements [90]. Reporting both within-subject and between-subject variance is essential for a comprehensive understanding of glucose dynamics. However, given the exploratory nature of this study and the limited sample size, the primary focus was placed on between-subject comparisons. These included the presentation of average glucose values and related metrics, along with their corresponding SDs. While the potential value of calculating within-subject variance was acknowledged during the analytical process, the fact that the challenges were repeated only twice raised concerns that reliable estimation of within-subject variance would be of limited utility and could potentially yield misleading results. For these reasons, within-subject variance was not included in the current analysis. Furthermore, the main objective of this study was to assess the practicability of the intervention using basic statistical tests to validate its feasibility and to inform the design of future study protocols.

### Conclusion

In this study, we utilized metrics to describe individual glucose responses to specific, standardized interventions, including food intake, physical activity, and stress, introducing an innovative method to quantify GRTB. To our knowledge, this is the first study to examine the relationship between stress and glucose levels in healthy, young adults using CGM. The data collected in this context can serve as valuable reference data for future investigations, which should aim to include larger sample sizes to validate these findings. Beyond the management of metabolic diseases, these metrics may also be applicable to healthy individuals for monitoring of healthy lifestyle.

## Supporting information

S1 Protocol Study protocol. (DOCX)

S1 Appendix TREND Checklist. (S1_Appendix.DOCX)

S1 Fig CGM satisfaction survey. (S1_Fig.DOCX)

S1 Table Composition of food for food challenges. (S1_Table.DOCX)

S2 Fig Survey on usefulness of various functions of CGM devices. (S2_Fig.DOCX)

S2 Table Instructions of food intake. (S2_Table.DOCX)

S3 Fig WHO-5-wellbeing Index. (S3_Fig.DOCX)

S3 Table Instructions for standardized sport session. (S3_Table.DOCX)

S4 Table Overall CGM metrics. (S4_Table.DOCX)
