# Supplementary material for: Continuous Glucose Monitoring under standardised conditions regarding diet, exercise and stress in Healthy Young People (CGM-HYPE study): An exploratory clinical trial
Source: PLOS Digit Health. 2025 Nov 14;4(11):e0001087. doi: 10.1371/journal.pdig.0001087 (PMC12617953; doi:10.1371/journal.pdig.0001087)
Supplement: S1 Protocol — (DOCX) [file pdig.0001087.s001.docx]

| 1. **Title** | „Continuous glucose monitoring under standardised conditions regarding diet, exercise and stress in healthy young people (CGM-HYPE): An exploratory clinical trial” | |
| --- | --- | --- |
| 1. **Person responsible** | Florian Kinny  Heinrich Heine University Düsseldorf | Gender of the person responsible:  Female  Male  Divers |
| 1. **Institution** | Faculty of Mathematics and Natural Sciences  Institute for Clinical Pharmacy and Pharmacotherapy  Heinrich Heine University Düsseldorf  Universitätsstraße 1, 40225 Düsseldorf | |
| 1. **Background** | Type 2 diabetes mellitus (T2DM) is a chronic disease with a prevalence of 7.2 % in Germany, in which the body's glucose and insulin balance are disturbed [1]. The treatment of T2DM consists of lifestyle changes and the application of drugs that promote the sensitivity and release of insulin, the hormone required for glucose uptake. If poorly treated, the disease can lead to complications and damage to small vessels in the nerves, kidneys or eyes, which can result in nephropathy, neuropathy or even blindness. Prevention and well-managed treatment of T2DM are particularly important for improving patients' quality of life and reducing the burden on the healthcare system.  In the context of pharmaceutical counselling and therapy monitoring, today's pharmacists are increasingly confronted with digital tools and applications for T2DM. For example, in addition to telepharmacy (provision of pharmaceutical services such as remote counselling), new methods for continuous glucose monitoring in real time (CGM, continuous glucose monitoring) are now available under the term "digital health". These are able to replace the previously standard, but cumbersome and stressful blood glucose determination by means of blood sampling by the patient (SMBG, self-monitoring blood glucose) and at the same time generate significantly more data, which can improve glycaemic control and allow more comprehensive glucose regulation. CGM devices are also characterised by their ease of use and can be prescribed by medical staff if the costs are covered by statutory health insurance (SHI). They are also advertised to private customers. As qualified and easily accessible contact persons for patients, pharmacists must be able to advise on these new digital devices and interpret the data they collect.  Current CGM devices generate predetermined CGM metrics according to international consensus [2]. These metrics are calculations and mean values from data collected over 14 days. Individual glucose reactions to certain stimuli such as (standardised) food, exercise or stress are not taken into account in the algorithm for calculating the metrics, but could offer added value for early detection and prevention. Such glucose reactions need to be researched and then, if necessary, added to the existing metrics. | |
| 1. **Objectives** | This research aims to conduct a standardized analysis of CGM data obtained from healthy young individuals under controlled conditions, with a focus on individual glucose responses due to diet, sport and psychobiological stress, to establish these data as reference values | |
| 1. **Study design** | Exploratory, interventional, clinical trial (*Ethics committee register-Nr. 2023-2647*)  German register for clinical trial (DRKS): https://drks.de/search/de/trial/DRKS00032821 | |
| 1. **Study duration** | Each participating person has a time commitment of 14 days. Several participants can also take part in the study at the same time. | |
| 1. **Study population** | Healthy, young adults (n=10) | |
| 1. **Study procedure** | Participants are primarily recruited on the Heinrich Heine University Düsseldorf (HHUD) campus, at the Institute of Clinical Pharmacy and Pharmacotherapy, and through direct contact with acquaintances. They are provided with detailed participant information outlining the study's content and data handling procedures, and informed consent is obtained via signature. Following consent, fasting blood glucose levels are measured using a glucometer (Accu-Check® Guide, Roche Diabetes Care GmbH, Mannheim, Germany), an oral glucose tolerance test (2h-OGTT) is conducted, body mass index (BMI) is calculated, and waist circumference is measured. Eligible participants register pseudonymously with the LibreView® Data Management System to enable data collection. CGM systems (FreeStyle Libre 3®, Abbott Diabetes Care GmbH, Wiesbaden, Germany) are applied to the upper, non-dominant arm by participants under study staff supervision, following the manufacturer's instructions. The sensors are connected via Bluetooth to the Libre 3® app, pre-installed on participants' mobile devices, using the accounts created during registration. The study coordinator links all accounts through the LibreView® Cloud for centralized data access and later exports the data via this platform. The CGM systems, provided by the Institute of Clinical Pharmacy and Pharmacotherapy, are operational for 14 consecutive days after a one-hour automatic calibration. During the 14-day period, participants document their food intake (type, quantity, and timing), physical activity (type, duration, and intensity), sleep-wake patterns, and stress events using the app's note function. While wearing the CGM system, participants agree to complete nine standardized "challenges" designed to elicit glucose responses. All interventions are conducted under fasting conditions, with no food intake or physical activity for at least 4 hours before and after each challenge:  Table 1. Overview of the challenges to be completed   \| Nr. \| Category \| Challenge \| \| --- \| --- \| --- \| \|  \| Food \| Breakfast 1 (without additional fibre) \| \|  \| Food \| Breakfast 2 (with additional fibre) \| \|  \| Food \| Breakfast 2 (with additional fibre) \| \|  \| Food \| Vegetables (Frosta® Bio Gelbes Curry) \| \|  \| Food \| Vegetables (Frosta® Bio Gelbes Curry) \| \|  \| Food \| Pizza (Wagner® Margherita) \| \|  \| Sport \| Functional Fitness (Strength exercises with your own body weight) \| \|  \| Sport \| Crosstrainer (Endurance exercises, aerobic training) \| \|  \| Stress \| Fictitious job interview (Trier-Social-Stress-Test) \|   Participants will receive food provided by the Institute of Clinical Pharmacy and Pharmacotherapy, with detailed procedures for food preparation and intake outlined in the manuscript appendix (S1. Appendix: Food Challenges). The sports exercises, performed once, consist of a strength exercise using body weight and an endurance exercise. The sequence of functional fitness exercises is shown in detail in the manuscript’s appendix (S2. Appendix: Sport Challenges). The functional fitness sport challenge can be completed at home and takes approximately 40 minutes, while the endurance challenge involves a 30-minute session on an aerial bike, conducted under supervision in the gymnasium of the HHUD sports centre. During the stress test, participants undergo a simulated 4-minute job interview, for which they are given 5 minutes to prepare. Following the interview, they complete mental arithmetic tasks for 4 minutes. Saliva samples (Sarstedt Salivette®, Germany) are collected before the test, between tasks, immediately after the final task, and after a 30-minute relaxation phase. Cortisol levels (measured by Dresden LabService GmbH, Germany) are analysed to confirm stress induction. Participants also report their subjective stress levels using a visual analogue scale (VAS) before, twice during, and after the test. Except for the stress test, all interventions can be performed either at the institute or at home, provided they are conducted at the same time of day. Participants must complete each intervention on an empty stomach, refraining from food intake or physical activity for at least 4 hours before and after the intervention. After 14 days, the CGM sensor automatically deactivates and ceases glucose measurements. The sensor is then removed, and the application site is examined for skin irritation. The study coordinator retrieves all participant data from the LibreView® Cloud for analysis. At this point, the LibreView® accounts and app can be deleted. Finally, participants' satisfaction with the CGM system and their quality of life is determined using a survey. | |
| 1. **Teilnehmenden-Zeitleiste** | 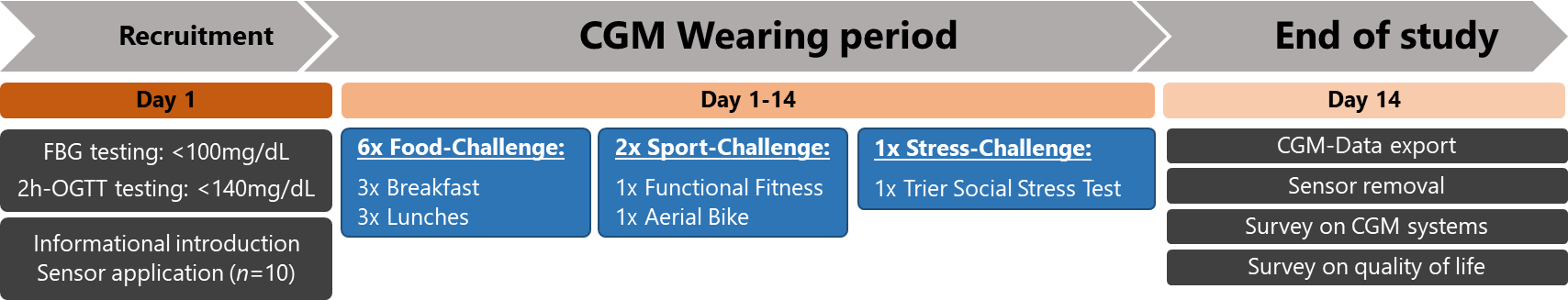  **Figure 1.** Study programme for one participant, FBG: Fasting Blood Glucose, OGTT: Oral Glucose Tolerance Test | |
| 1. **Inclusion criteria** | - > 18 & < 40 years old - Waist circumference (Male < 94 cm, Female < 80 cm) - Body-Mass-Index (BMI) > 18,5 & < 30 kg/m^2^ - Healthy according to own information (absence of heart or metabolic diseases, no intake of corresponding medication) - Fasting Blood Glucose < 100 mg/dL - Blood Glucose < 140 mg/dL two hours after onset of oral glucose tolerance test (2h-OGTT) - Mobile smartphone and willingness to use it as part of the study - Voluntary consent to participate in the interventional study - Voluntary consent to data protection in the interventional study | |
| 1. **Exclusion criteria** | - Failure to provide informed consent for participation in this interventional study - Failure to provide informed consent for data protection - Diagnosis of diabetes mellitus type I or II (self-reported) - Eating or thyroid dysfunction - Allergies to food or patches - Cognitive or physical limitations - Smoking - Pregnancy/breastfeeding - Taking vitamin C (possible interference with CGM system → masking of low values) [3] - Taking metabolic drugs (e.g. cortisol, antidiabetics, insulin, broncholytics, antihypertensives, immunomodulatory drugs) | |
| 1. **Study endpoints** | Glucose response to all nine challenges: Determination of metrics as measures of the glucose response for each of the nine challenges:   1. area under the glucose curve (AUC) from the start of the challenge to four hours after the challenge using trapezoid rule [(mg/dL)*min] 2. initial baseline (mean of the three consecutive glucose readings recorded before the start of the challenge) 3. c_max_: maximum glucose concentration [mg/dL] 4. t_max_: time to c_max_ [min] 5. glucose excursion (difference of the c_max_ and baseline) [mg/dL] 6. peak duration: time required for glucose levels to return to initial baseline (Glucose Recovery Time to Baseline (GRTB)) [min]   This metric is described with mean ± standard deviation of all participants as a descriptive measure for each individual challenge.  CGM metrics according to international consensus [2]   - Percent of the time in the target range (time in range, TIR 70-180 mg/dL) - Percent of the time below the target range (TBR < 70mg/dL) - Percent of time above the target range (Time above range, TAR > 180mg/dL) - Mean value of all glucose values (Mean glucose, mg/dL) - Glycaemic variability: Coefficient of variation (CoV) in % - Number of hypoglycemic events - The metrics are described as descriptive statistics (mean ± standard deviation and median with interquartile range (Q1-Q3)) - The metrics were examined with respect to gender and day-night time periods.   Survey on system satisfaction & quality of life | |
| 1. **Adverse events** | The use of CGM systems is generally not associated with significant health risks. Sensor application is a minimally invasive procedure, although the puncture site may serve as a potential entry point for pathogens. This risk can be mitigated through proper preparation of the skin and correct sensor application, for which instructional videos and detailed guidelines are provided. Skin irritation under the sensor may occur due to factors such as sweating or friction.  To prevent the sensor from detaching during physical activity, fixation plasters can be applied as needed. The sensor is adhered to the skin using acrylate-based adhesives, which may cause allergic reactions in some individuals. In such cases, the manufacturer recommends placing a hydrocolloid blister plaster without acrylates between the skin and the sensor to minimize allergic responses. Data protection risks are minimized through pseudonymization of the collected data. Pseudonymized personal data is stored separately from glucose data on secure servers located in Germany (Frankfurt am Main), ensuring that no direct link to individual participants can be established. The allocation key used for anonymization is destroyed following the completion of the "psychological stress" challenge, further safeguarding participant confidentiality. | |
| 1. **Data management** | The data collected by the CGM system is stored either locally on the participant's mobile device or on the LibreView® Cloud, operated by Abbott Diabetes Care GmbH, using AWS servers located in Frankfurt am Main. The cloud storage complies with GDPR regulations and employs AES 256-bit encryption for data security. While local storage does not require prior registration, cloud storage necessitates the creation of a LibreView Data Management System account. This account enables data export and graphical analysis, offering significant advantages for subsequent analyses. Each participant creates their own LibreView Data Management System account, which allows access to the LibreView® Cloud for glucose data storage. The data is pseudonymized, and the required registration details include residence, first and last name, date of birth, and an email address. To ensure anonymity, participants may use fictitious names and dates of birth, but the place of residence must be set to Germany to guarantee data storage on the Frankfurt-based AWS server. By using pseudonymous data, it is not possible to identify individual participants. After registration, participants can access and export their own data via the cloud. Additionally, all participants are grouped into a "practice" database created by the study coordinator within LibreView®, enabling centralized access to the collected data for analysis. Once data collection and export are complete, the practice database is deleted. Participants may also delete their LibreView Data Management System accounts after the study concludes. Participants can document their lifestyle using the notes function in the Libre 3® app, including details such as food intake, physical activity, and stress levels. All entries in the app are synchronized with the glucose data stored in the LibreView® Cloud. Upon account deletion, all associated data, including glucose readings and lifestyle documentation, is irrevocably deleted. | |
| 1. **References** | \| [1] \| C. Heidemann und C. Scheidt-Nave, „Prävalenz, Inzidenz und Mortalität con Diabetes melliltus bei Erwachsenen in Deutschland,“ *Journal of Health Monitoring,* pp. 105-129, 13 09 2017. \| \| --- \| --- \| \| [2] \| T. Battelino et al, „Continuous glucose monitoring and metrics for clinical trials: an international consensus statement,“ *Lancet Diabetes Endocrinol,* pp. 42-57, Jan 2023. \| \| [3] \| Abbott, „www.freestylelibre.com,“ 2021. [Online]. Available: https://www.freestyle.abbott/us-en/safety-information.html. [Zugriff am 18 10 2023]. \| | |
