# Supplementary material for: Continuous Glucose Monitoring under standardised conditions regarding diet, exercise and stress in Healthy Young People (CGM-HYPE study): An exploratory clinical trial
Source: PLOS Digit Health. 2025 Nov 14;4(11):e0001087. doi: 10.1371/journal.pdig.0001087 (PMC12617953; doi:10.1371/journal.pdig.0001087)
Supplement: S1 Table — (S1_Table.DOCX) [file pdig.0001087.s004.docx]

|  | Description | Carbo-  hydrates [g] | Protein [g] | Fat [g] | Fibers [g] | Calories  [kcal] | n |
| --- | --- | --- | --- | --- | --- | --- | --- |
| Food 1 | Carrot, tomatoes, cucumber, Yogurt with berries | 30 | 19 | 12 | 0 | 384 | 1 |
| Food 2 | Carrot, tomatoes, cucumber, Yogurt with berries + extra fibre | 50 | 19 | 12 | 15 | 414 | 2 |
| Food 3 | Potato dish with vegetables in a curry sauce | 34 | 11 | 13 | 11 | 315 | 2 |
| Food 4 | Pizza Margherita | 81 | 33 | 27 | 6 | 706 | 1 |
