# Supplementary material for: Continuous Glucose Monitoring under standardised conditions regarding diet, exercise and stress in Healthy Young People (CGM-HYPE study): An exploratory clinical trial
Source: PLOS Digit Health. 2025 Nov 14;4(11):e0001087. doi: 10.1371/journal.pdig.0001087 (PMC12617953; doi:10.1371/journal.pdig.0001087)
Supplement: S2 Fig — (S2_Fig.DOCX) [file pdig.0001087.s005.docx]

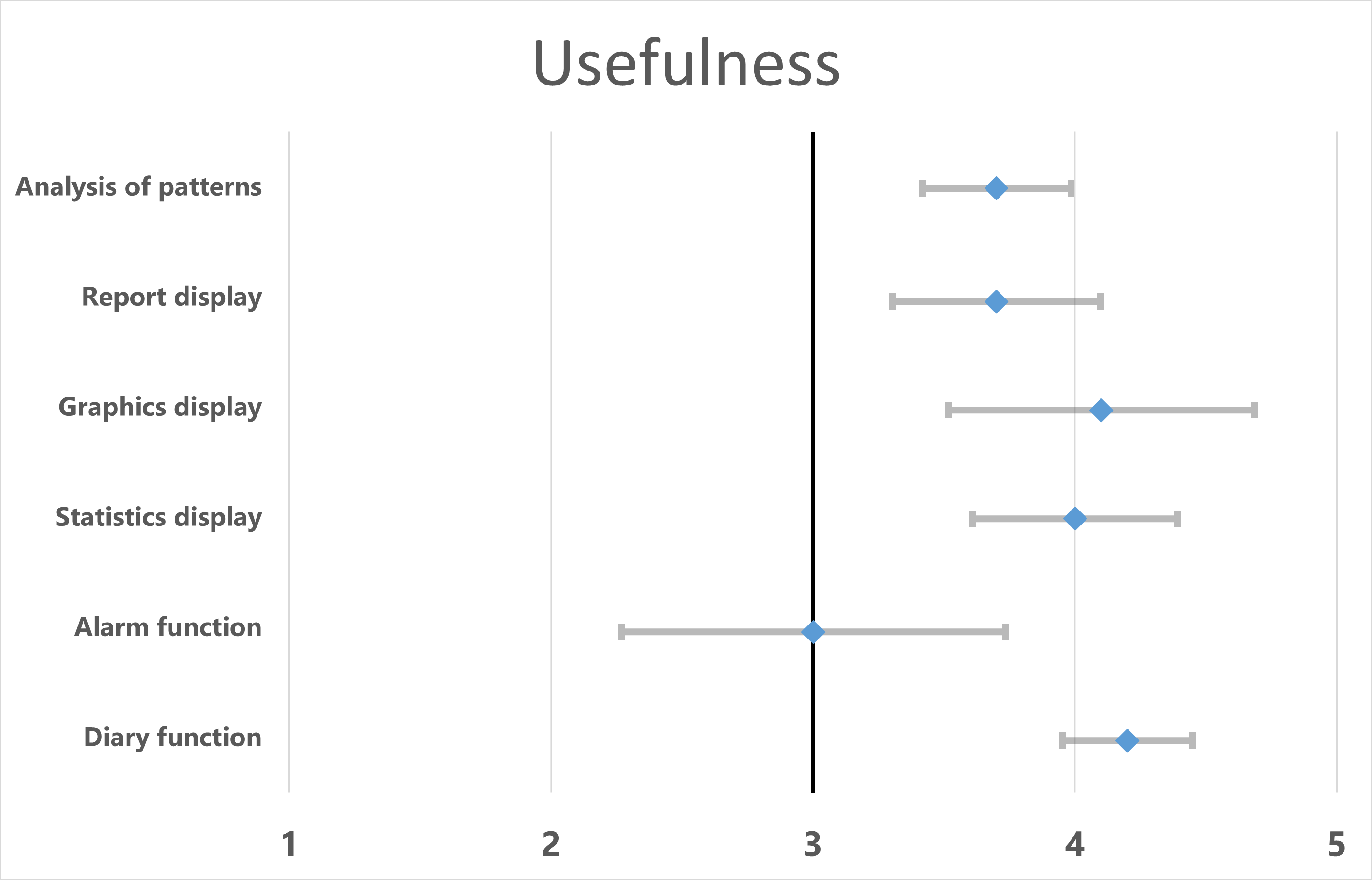


*S2 Fig. Forest plot of the mean values with 05% confidence interval for the usefulness of the various functions of the continuous glucose monitoring (CGM) system app, n = 10 (1=”very useful”,5=”not useful at all”)*
