# Supplementary material for: Continuous Glucose Monitoring under standardised conditions regarding diet, exercise and stress in Healthy Young People (CGM-HYPE study): An exploratory clinical trial
Source: PLOS Digit Health. 2025 Nov 14;4(11):e0001087. doi: 10.1371/journal.pdig.0001087 (PMC12617953; doi:10.1371/journal.pdig.0001087)
Supplement: S2 Table — (S2_Table.DOCX) [file pdig.0001087.s006.docx]

| Breakfast 1 (n=1) | |
| --- | --- |
| Food | Carrot, tomatoes, cucumber, Yogurt with berries |
| Ingredients | - - - 100g blueberries     - 50g carrot     - 100g cucumber     - 50 cherry tomatoes     - 150g natural yogurt |
| Preparation | - Stir the yogurt once with a spoon - Add the washed berries to the yoghurt - Now mix these ingredients together - The vegetables are already weighed and chopped |
| Ingestion | - - - 7 – 9 am     - Order of ingestion: vegetables first, later yogurt     - With still water if necessary |
| Nutritional values | |
| Carbohydrates | 30 g |
| Protein | 19 g |
| Fat | 12 g |
| Fiber | 0 g |
| Calories | 384 kcal |
| Breakfast 2 (n=2) | |
| Food | Carrot, tomatoes, cucumber, Yogurt with berries + extra fibre |
| Ingredients | - - - 100g blueberries     - 50g carrot     - 100g cucumber     - 50 cherry tomatoes     - 150g natural yogurt     - 4 tablespoons spelt flakes     - 2 tablespoons ground linseed |
| Preparation | - Stir the yogurt once with a spoon - Add the washed berries to the yoghurt - Now mix these ingredients together - Sprinkle all the spelt flakes and ground linseed over the yoghurt and berry mix - The vegetables are already weighed and chopped |
| Ingestion | - - - 7 – 9 am     - Order of ingestion: vegetables first, later yogurt     - With still water if necessary |
| Nutritional values | |
| Carbohydrates | 50 g |
| Protein | 19 g |
| Fat | 12 g |
| Fiber | 15 g |
| Calories | 414 kcal |
| Lunch 1 (n=2) | |
| Food | Potato dish with vegetables in a curry sauce |
| Ingredients | - - - Cauliflower     - French beans     - Mushrooms     - Potatoes     - Garlic     - carrot     - leek     - Corn and potato starch     - Tomatoes & tomato juice     - Coconut milk |
| Preparation | **In the pan**   - Pour the contents into the pan with three tablespoons of water - Heat over a medium heat - Cook for eight to nine minutes over a medium heat until the desired sauce consistency is reached - Stir occasionally   **In the microwave**   - Place the contents with a tablespoon of water in a microwave-safe container without a lid - Cook at 600 watts for around seven minutes - Stir halfway through and at the end |
| Ingestion | - - - 1 – 3 pm     - With still water if necessary |
| Nutritional values | |
| Carbohydrates | 34,2 g |
| Protein | 10,8 g |
| Fat | 12,6 g |
| Fiber | 10,8 g |
| Calories | 315 kcal |
| Lunch 2 (n=1) | |
| Food | Pizza margherita |
| Ingredients | - - - Wheat flour     - chopped peeled tomatoes (26%)     - firm mozzarella (25%)     - water     - rapeseed oil     - yeast     - salt     - sugar     - dried wheat yeastd ough |
| Preparation | **In the oven**   - top/bottom heat: 230 °C fan oven - 200 °C Preheat the oven to the temperature indicated opposite - Leave the pizza in the freezer while the oven preheats - remove the foil - place the pizza on the middle shelf of the oven - bake the pizza at 230 °C top/bottom heat for at least 10 minutes - The cheese should be melted and the edges golden brown - allow the pizza to cool for at least 1 minute before eating |
| Ingestion | - - - 1 – 3 pm     - With still water if necessary |
| Nutritional values | |
| Carbohydrates | 86,1 g |
| Protein | 32,6 g |
| Fat | 26,6 g |
| Fiber | 6,6 g |
| Calories | 727 kcal |
