# Supplementary material for: Continuous Glucose Monitoring under standardised conditions regarding diet, exercise and stress in Healthy Young People (CGM-HYPE study): An exploratory clinical trial
Source: PLOS Digit Health. 2025 Nov 14;4(11):e0001087. doi: 10.1371/journal.pdig.0001087 (PMC12617953; doi:10.1371/journal.pdig.0001087)
Supplement: S3 Fig — (S3_Fig.DOCX) [file pdig.0001087.s007.docx]

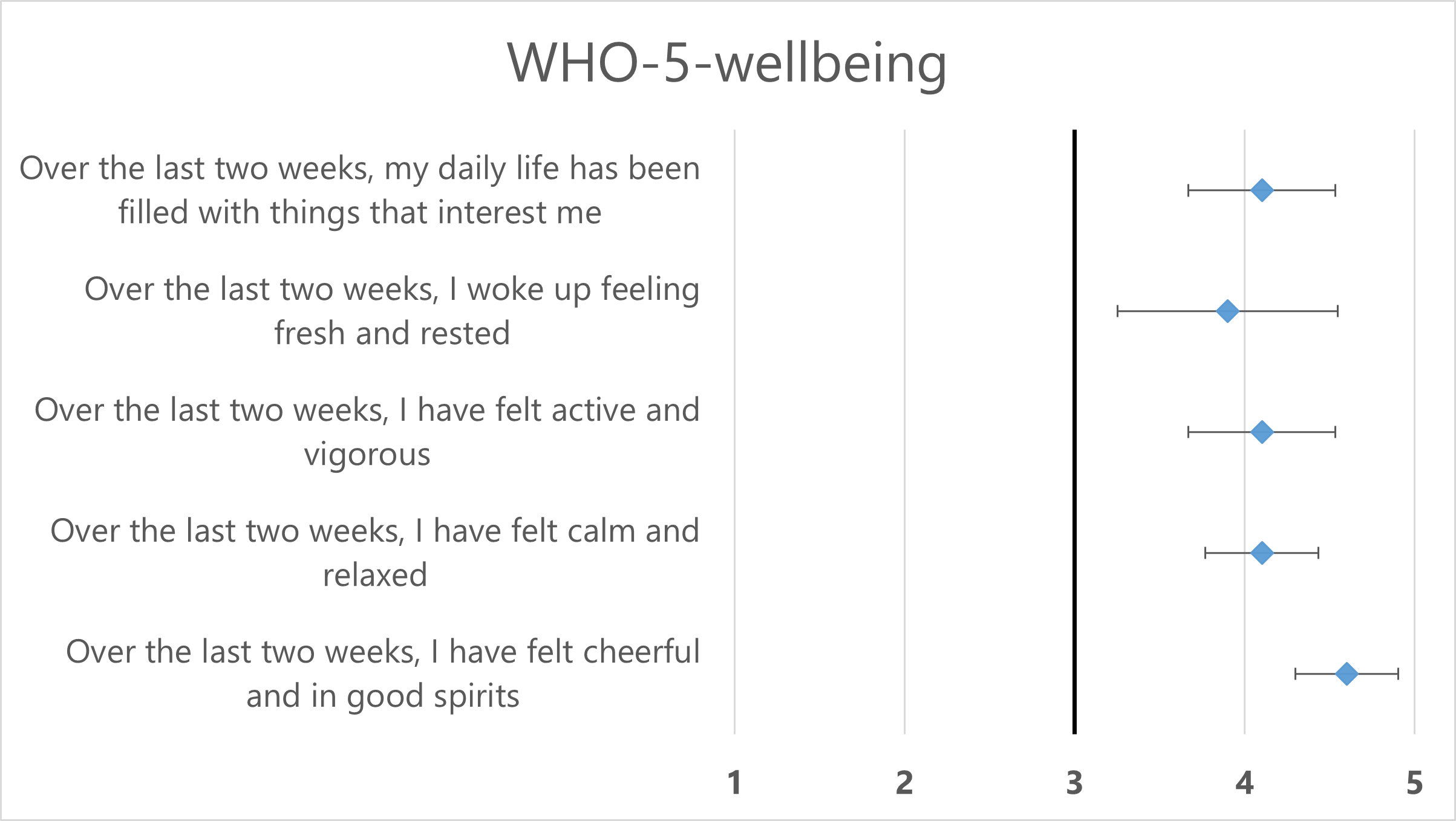


*S3 Fig. Forest plot of the mean values with 95% confidence interval of the WHO Five Well Being Index (WHO-5), n = 10 (1=”at no time”,5=”all of the time”)*
