# Supplementary material for: Continuous Glucose Monitoring under standardised conditions regarding diet, exercise and stress in Healthy Young People (CGM-HYPE study): An exploratory clinical trial
Source: PLOS Digit Health. 2025 Nov 14;4(11):e0001087. doi: 10.1371/journal.pdig.0001087 (PMC12617953; doi:10.1371/journal.pdig.0001087)
Supplement: S3 Table — (S3_Table.DOCX) [file pdig.0001087.s008.docx]

| **Functinal Fitness**  **(30 second-break after each exercise)** | |
| --- | --- |
| Jumping Jacks | - 40 seconds |
| Raising knees | - rotate from the inside to the outside or vice versa - 12x each knee |
| Heels up | - Stand upright with your feet hip-width apart - Slowly lift your heels off the ground - Rise onto the balls of your feet - Hold this position for a moment to engage your calf muscles. - Gradually lower your heels back down to the starting position - Maintain control throughout the movement - 20 seconds each side |
| Lunges | - Place legs shoulder-width apart - Leg out at a 90° angle - Knee must not point over toes - Step back to starting position - Then with other leg - 12 repetitions |
| Sit-ups | - Supine position with bent legs - Arms to the temples or chest - Do not pull on the head) - Exhale when going up - Breathe in as you go down - Lower back remains on the floor - 2x 15 repetitions |
| Super(wo)man | - Lie on your stomach - Arms stretched out in front - Raise arms and legs & wiggle - 2x 40 seconds |
| Squat Jumps | - Legs slightly wider than shoulder width - Start with a squat - Do a jump from this position - Note: Always bend your knees over your ankles, not inwards - 2x 12 repetitions |
| Planks | - Lean on toes and elbows - Back straight - Buttocks remain in line with shoulders and heels - Hold for 2x 40 seconds |
| Hip Thrust | - Lie on your back - Bend legs, arms outstretched from body - From this position, push the pelvis upwards - Hold in the top position for three seconds - 2x 12 repetitions |
| Four-footed leg raise | - Get into the four-footed position - Weight is distributed on the knees and hands - Knees under hips, hands under shoulders - Raise your leg and point the sole towards the ceiling - Rock in the top position for 30 seconds - Alternate each leg twice |
| Push-ups | - Sit on your hands and knees - Tense your core - Knees, buttocks, stomach & shoulders form a line - Exhale while pushing up - 2x 8 - 12 repetitions |
| Rowing with a water bottle | - Slightly bent legs - Wide stance - Lean forward with a straight back - Hold filled bottle in one hand - Raise both arms, pressing shoulder blades together - Lower arms, transfer bottle to other hand - 2x 15 repetitions |
| Four-footed stance | - Get into the four-footed stance - Weight is distributed on the knees and hands - Knees under hips, hands under shoulders - Extend arm and opposite leg - Then pull your elbow towards your knee, making a hump - Then switch sides - 2x 12 repetitions |
