# Supplementary material for: Continuous Glucose Monitoring under standardised conditions regarding diet, exercise and stress in Healthy Young People (CGM-HYPE study): An exploratory clinical trial
Source: PLOS Digit Health. 2025 Nov 14;4(11):e0001087. doi: 10.1371/journal.pdig.0001087 (PMC12617953; doi:10.1371/journal.pdig.0001087)
Supplement: S4 Table — (S4_Table.DOCX) [file pdig.0001087.s009.docx]

|  | 24 H | Female | Male |
| --- | --- | --- | --- |
| **Overall glucose data, mean ± SD** | | | |
| Mean glucose, mg/dL | 106.24 ± 6.46 | 103.09 ± 6.43 | 110.96 ± 2.61 |
| Glucose SD, mg/dL | 16.56 ± 3.09 | 16.52 ± 2.52 | 16.62 ± 4.24 |
| Glucose CoV, % | 15.65 ± 3.23 | 16.14 ± 3.22 | 14.93 ± 3.59 |
| MAGE, mg/dL | 44.2 ± 9.01 | 44.35 ± 8.54 | 43.98 ± 12.23 |
| AUC, (mg/dL)*min | 2130135.5 ± 131723.88 | 2065092.08 ± 129944.57 | 2227700.62 ± 52513.41 |
| **% of sensor values, median (IQR)** | | | |
| % time > 250 mg/dL | 0.00%  (0.00% - 0.00%) | 0.00%  (0.00% - 0.00%) | 0.00%  (0.00% - 0.00%) |
| % time > 180 mg/dL | 0.09%  (0.04% - 0.5%) | 0.08%  (0.04% - 0.17%) | 0.36%  (0.08% - 0.67%) |
| % time > 160 mg/dL | 0.74%  (0.32% - 1.99%) | 0.62%  (0.32% - 1.24%) | 1.61%  (0.66% - 2.51%) |
| % time > 140 mg/dL | 3.88%  (2.54% - 7.35%) | 2.9%  (2.54% - 4.29%) | 6.83%  (4.82% - 8.35%) |
| % time > 120 mg/dL | 16.64%  (11.18% - 21.56%) | 12.99%  (11.18% - 17.82%) | 24.48%  (19.13% - 27.05%) |
| % time < 70 mg/dL | 0.00%  (0.00% - 0.14%) | 0.01%  (0.00% - 0.14%) | 0.00%  (0.00% - 0.05%) |
| % time < 60 mg/dL | 0.00%  (0.00% - 0.00%) | 0.00%  (0.00% - 0.00%) | 0.00%  (0.00% - 0.01%) |
| % time < 54 mg/dL | 0.00%  (0.00% - 0.00%) | 0.00%  (0.00% - 0.00%) | 0.00%  (0.00% - 0.00%) |
| % time in range 70 - 180 mg/dL | 99.76%  (99.38% - 99.92%) | 99.76%  (99.49% - 99.89%) | 99.64%  (99.28% - 99.93%) |
| % time in range 70 - 160 mg/dL | 99.25%  (97.69% - 99.54%) | 99.36%  (98.21% - 99.54%) | 98.39%  (97.44% - 99.34%) |
| % time in range 70 - 140 mg/dL | 95.58%  (91.82% - 97.46%) | 97.09%  (93.02% - 97.46%) | 93.17%  (91.6% - 95.18%) |
| % time in range 70 - 120 mg/dL | 82.24%  (78.44% - 87.51%) | 84.62%  (81.61% - 87.51%) | 75.52%  (72.9% - 80.87%) |
| % time in range 180 - 250 mg/dL | 0.12%  (0.04% - 0.54%) | 0.09%  (0.04% - 0.17%) | 0.40%  (0.11% - 0.70%) |
| % time in range 54 - 70 mg/dL | 0.02%  (0.00% - 0.21%) | 0.08%  (0.01% - 0.21%) | 0.00%  (0.00% - 0.07%) |
| n hypoglycemic event | 0 (0.00%) | 0 (0.00%) | 0 (0.00%) |
| **Daytime glucose data, mean ± SD** | | | |
| Mean glucose, mg/dL | 107.57 ± 7.22 | 104.17 ± 6.83 | 112.68 ± 4.54 |
| Glucose SD, mg/dL | 17.13 ± 3.54 | 17.35 ± 2.67 | 16.81 ± 5.05 |
| Glucose CoV, % | 15.99 ± 3.55 | 16.77 ± 3.32 | 14.82 ± 4.06 |
| **% of sensor values, median (IQR)** | | | |
| % time > 250 mg/dL | 0.00%  (0.00% - 0.00%) | 0.00%  (0.00% - 0.00%) | 0.00%  (0.00% - 0.02%) |
| % time > 180 mg/dL | 0.2%  (0.05% - 0.67%) | 0.18%  (0.05% - 0.27%) | 0.48%  (0.1% - 0.89%) |
| % time > 160 mg/dL | 0.98%  (0.81% - 2.65%) | 0.83%  (0.81% - 1.66%) | 2.14%  (0.86% - 3.35%) |
| % time > 140 mg/dL | 4.64%  (3.61% - 9.32%) | 3.66%  (3.61% - 5.14%) | 8.14%  (4.84% - 11.15%) |
| % time > 120 mg/dL | 17.68%  (10.65% - 23.92%) | 13.21%  (10.65% - 19.86%) | 27.14%  (17.71% - 33.88%) |
| % time < 70 mg/dL | 0.00%  (0.00% - 0.00%) | 0.00%  (0.00% - 0.00%) | 0.00%  (0.00% - 0.02%) |
| % time < 60 mg/dL | 0.00%  (0.00% - 0.00%) | 0.00%  (0.00% - 0.00%) | 0.00%  (0.00% - 0.00%) |
| % time < 54 mg/dL | 0.00%  (0.00% - 0.00%) | 0.00%  (0.00% - 0.00%) | 0.00%  (0.00% - 0.00%) |
| % time in range 70 - 180 mg/dL | 99.88%  (99.18% - 99.97%) | 99.94%  (99.38% - 99.97%) | 99.52%  (99.09% - 99.9%) |
| % time in range 70 - 160 mg/dL | 99.04%  (96.91% - 99.4%) | 99.3%  (97.63% - 99.4%) | 97.86%  (96.63% - 99.14%) |
| % time in range 70 - 140 mg/dL | 95.31%  (89.58% - 96.57%) | 96.49%  (91.46% - 96.57%) | 91.86%  (88.83% - 95.16%) |
| % time in range 70 - 120 mg/dL | 81.62%  (76.09% - 88.74%) | 84.51%  (79.78% - 88.74%) | 72.81%  (66.04% - 82.29%) |
| % time in range 180 - 250 mg/dL | 0.23%  (0.06% - 0.72%) | 0.2%  (0.06% - 0.27%) | 0.53%  (0.15% - 0.93%) |
| % time in range 54 - 70 mg/dL | 0.03%  (0.00% - 0.1%) | 0.08%  (0.02% - 0.1%) | 0.00%  (0.00% - 0.04%) |
| **Nighttime glucose data, mean ± SD** | | | |
| Mean glucose, mg/dL | 102.26 ± 5.72 | 99.87 ± 5.52 | 105.84 ± 4.33 |
| Glucose SD, mg/dL | 12.98 ± 2.96 | 12.83 ± 3.48 | 13.21 ± 2.48 |
| Glucose CoV, % | 12.75 ± 3.21 | 12.97 ± 4.03 | 12.43 ± 1.88 |
| **% of sensor values, median (IQR)** | | | |
| % time > 250 mg/dL | 0.00%  (0.00% - 0.00%) | 0.00%  (0.00% - 0.00%) | 0.00%  (0.00% - 0.00%) |
| % time > 180 mg/dL | 0.00%  (0.00% - 0.00%) | 0.00%  (0.00% - 0.00%) | 0.00%  (0.00% - 0.00%) |
| % time > 160 mg/dL | 0.00%  (0.00% - 0.00%) | 0.00%  (0.00% - 0.00%) | 0.00%  (0.00% - 0.05%) |
| % time > 140 mg/dL | 0.89%  (0.15% - 1.88%) | 0.89%  (0.32% - 1.84%) | 0.79%  (0.00% - 2.65%) |
| % time > 120 mg/dL | 8.38%  (6.72% - 12.38%) | 8.38%  (6.74% - 10.85%) | 11.51%  (6.9% - 18.1%) |
| % time < 70 mg/dL | 0.00  (0.00% - 0.08%) | 0.00%  (0.00% - 0.08%) | 0.00%  (0.00% - 0.12%) |
| % time < 60 mg/dL | 0.00%  (0.00% - 0.00%) | 0.00%  (0.00% - 0.00%) | 0.00%  (0.00% - 0.02%) |
| % time < 54 mg/dL | 0.00%  (0.00% - 0.00%) | 0.00%  (0.00% - 0.00%) | 0.00%  (0.00% - 0.00%) |
| % time in range 70 - 180 mg/dL | 100%  (99.9% - 100%) | 99.95%  (99.9% - 100%) | 100%  (99.88% - 100%) |
| % time in range 70 - 160 mg/dL | 99.9%  (99.83% - 100%) | 99.9%  (99.9% - 99.98%) | 99.9%  (99.73% - 100%) |
| % time in range 70 - 140 mg/dL | 99.06%  (97.67% - 99.6%) | 99.1%  (97.72% - 99.6%) | 98.96%  (97.35% - 99.63%) |
| % time in range 70 - 120 mg/dL | 90.27%  (85.22% - 93.19%) | 90.47%  (87.62% - 93.19%) | 88.24%  (81.9% - 92.73%) |
| % time in range 180 - 250 mg/dL | 0.00%  (0.00% - 0.00%) | 0.00%  (0.00% - 0.00%) | 0.00%  (0.00% - 0.00%) |
| % time in range 54 - 70 mg/dL | 0.00%  (0.00% - 0.08%) | 0.00%  (0.00% - 0.08%) | 0.00%  (0.00% - 0.15%) |

*MAGE = Mean Amplitude of Glycaemic Excursions^a^, CoV = Coefficient of variation, SD = Standard deviation, IQR = Interquartilrange, mg = milligram, dL = deciliter*

*^a^ defined as the arithmetic mean of the absolute differences between consecutive glucose peaks and nadirs that exceed one standard deviation of the mean glucose value, and is used as a metric for glucose variability*
